# Supplementary material for: Molecular arrangements in the first monolayer of Cu-phthalocyanine on In2O3(111)
Source: J Mater Chem C Mater. 2025 Jul 10;13(34):17650–61. doi: 10.1039/d5tc01394a (PMC12303158; doi:10.1039/d5tc01394a)
Supplement: TC-013-D5TC01394A-s001 [file TC-013-D5TC01394A-s001.pdf]

Supplementary Information (SI) for Journal of Materials Chemistry C.
This journal is © The Royal Society of Chemistry 2025

Supplementary Information

Molecular Arrangements in the First Monolayer of Cu-Phthalocyanine on $In_2O_3(111)$

Matthias Blatnik[1,2], Fabio Calcinelli[3], Andreas Jeindl[3], Moritz Eder[1], Michael Schmid[1], Jan Cechal[2], Ulrike Diebold[1], Peter Jacobson[1,4], Oliver T. Hofmann[3] and Margareta Wagner[1*]

[1] *Institute of Applied Physics, TU Wien, 1040 Vienna, Austria*
[2] *CEITEC – Central European Institute of Technology, Brno University of Technology, 612 00 Brno, Czech Republic*
[3] *Institute of Solid State Physics, Graz University of Technology, 8010 Graz, Austria*
[4] *School of Mathematics and Physics, University of Queensland, 4068 St. Lucia, Australia*

[*] Corresponding author: margareta.wagner@tuwien.ac.at

1. Experimental

- **Characterization of the stoichiometric $In_2O_3(111)$ surface**

The experiments were conducted on an $In_2O_3(111)$ single crystal with triangular shape and a surface area of ≈3 mm^2. The miscut angle of the sample from the (111) plane is ≈3°. Figure S1 shows the sample mounted onto an Omicron sample plate. The In_2O_3 crystal is yellow-transparent and the edges of this particular crystal are sloped by 60°. The LEED pattern of the pristine surface reflects the 3-fold symmetry of the atomic structure.

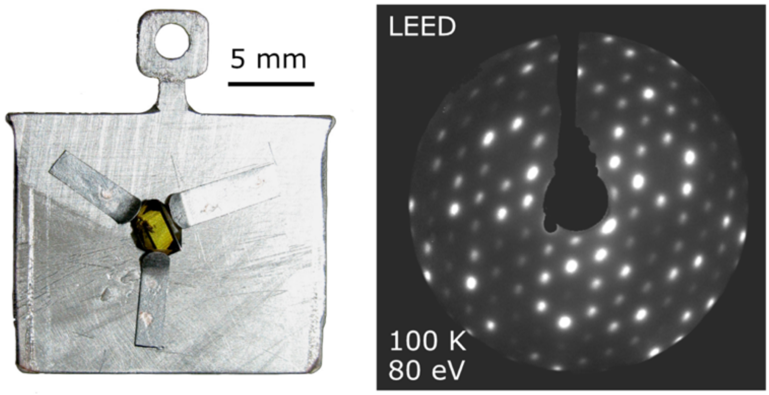

Figure S1: The $In_2O_3(111)$ single crystal used in this work and its LEED pattern.

The quality of the $In_2O_3(111)$ surface prior to CuPc deposition was investigated with STM, see Figure S2. The sample surface features terraces separated by steps of single-layer height (≈290 pm) with irregular step edges. Small islands, which grow during the oxidation (see methods section in the main text), and Argon bubbles (labelled with an asterisk *) related to the sample

cleaning by sputtering with Ar^+ ions are visible. The most common impurity encountered in STM is water (arrows in Figure S2(c) indicate single dissociated water molecules) adsorbing at temperatures <200 °C from the residual gas [1]. To desorb the water species, and also to provide sufficient energy for the CuPc molecules to diffuse, the sample is heated to 200 °C after CuPc deposition.

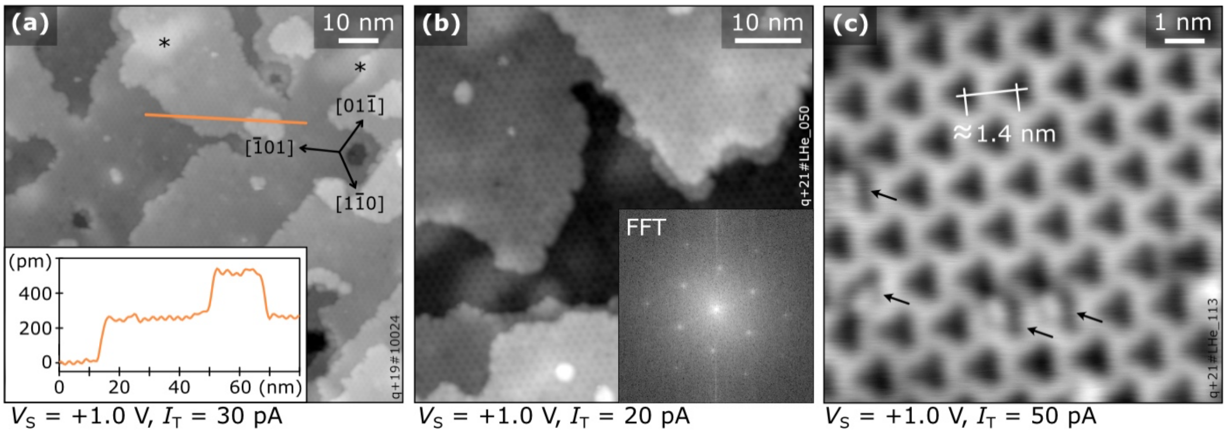

Figure S2: The stoichiometric the $In_2O_3(111)$ surface, constant-current empty-states STM. (a) Overview with a height profile across two steps, (b) more detailed view and its fast-Fourier transform (FFT) indicating the hexagonal lattice, and (c) detailed view featuring the typical contrast with dark triangles due to different densities of states associated with the 5- and 6-fold coordinated In atoms present at the surface [2]. Isolated water molecules [1] are marked by arrows.

The $In_2O_3(111)$ surface is an n-type semiconductor, with a band gap of almost 3 eV and the Fermi level positioned at the bottom of the conduction band. A scanning tunneling spectroscopy (STS) spectrum of the bare surface is shown in Figure S3. When imaging the surface with STM, empty states are probed.

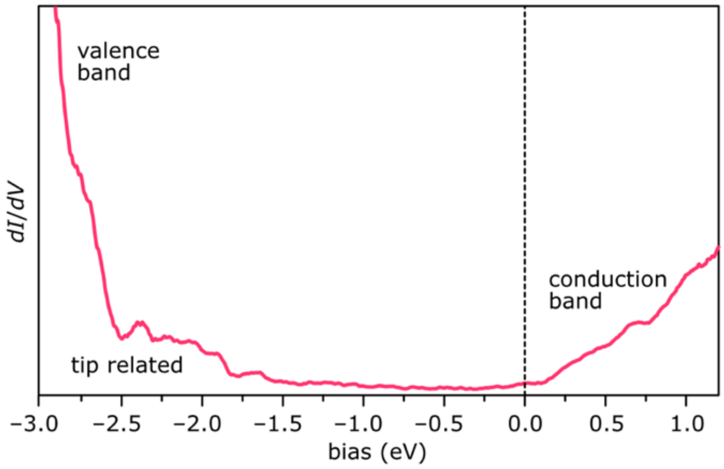

Figure S3: Local density of states (DOS) of the $In_2O_3(111)$ surface measured by STS with a metallic tip using a lock-in amplifier at 113 Hz and 10 mV_{pp} modulation voltage. Note that non-reproducible tip-related states are visible in the band gap close to the valence band.

- **Determining the adsorption site of CuPc**

To determine the adsorption site of CuPc with high accuracy but without atomic resolution of the surface in the presence of CuPc molecules, the following procedure was used: (1) The correlation of the pattern observed in empty-states STM images of the bare surface (dark triangles) to the atomic structure needs to be known. This is the case from simultaneous constant-height STM/AFM imaging with atomic resolution in the AFM channel, see Extended Data in Ref. [3]. Thus, the contrast of empty-states STM images can be clearly related to the atomic structure; this is also corroborated by DFT calculations. (2) To identify the adsorption site of the CuPc molecules, this correlation is utilized by aligning the simultaneously-acquired STM and AFM images of CuPc on $In_2O_3(111)$ with the known atomic structure of the substrate, as demonstrated in Figures S4 and S5. The accuracy of this approach is sufficient to locate the Cu ion near the oxygen atom labeled as O(γ) and to determine the molecule's rotational orientation, as discussed in the manuscript.

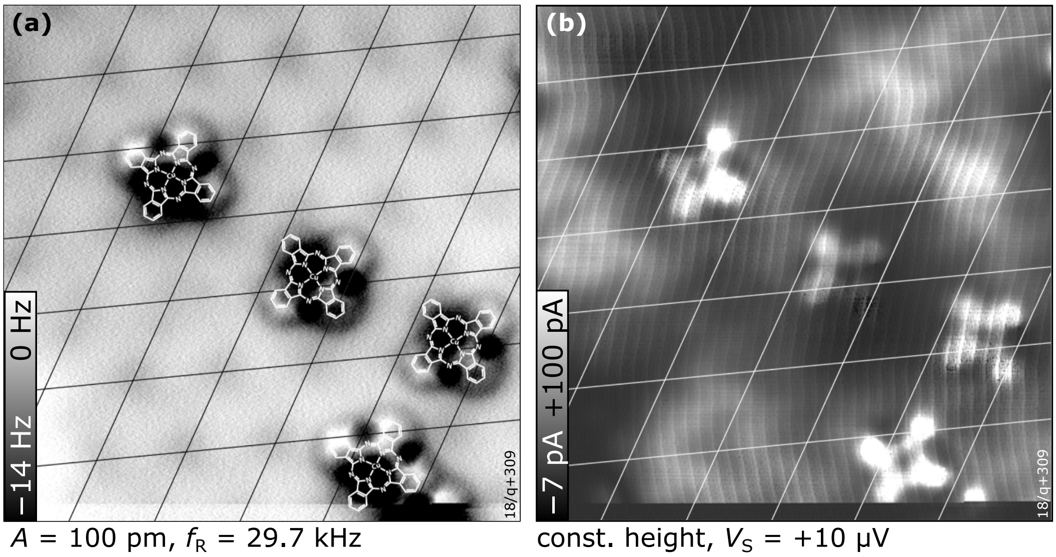

Figure S4: Identification of the adsorption site of the CuPc molecules by utilizing the known correlation between STM/AFM contrast and the atomic structure of the $In_2O_3(111)$ surface. Frame (b) shows the tunneling current acquired simultaneously with the AMF image. Due to the very small bias voltage (nominally zero), strong interference signals at the line frequency and its overtones are visible; nevertheless, the dark triangular features at the corners of the surface unit cells are visible.

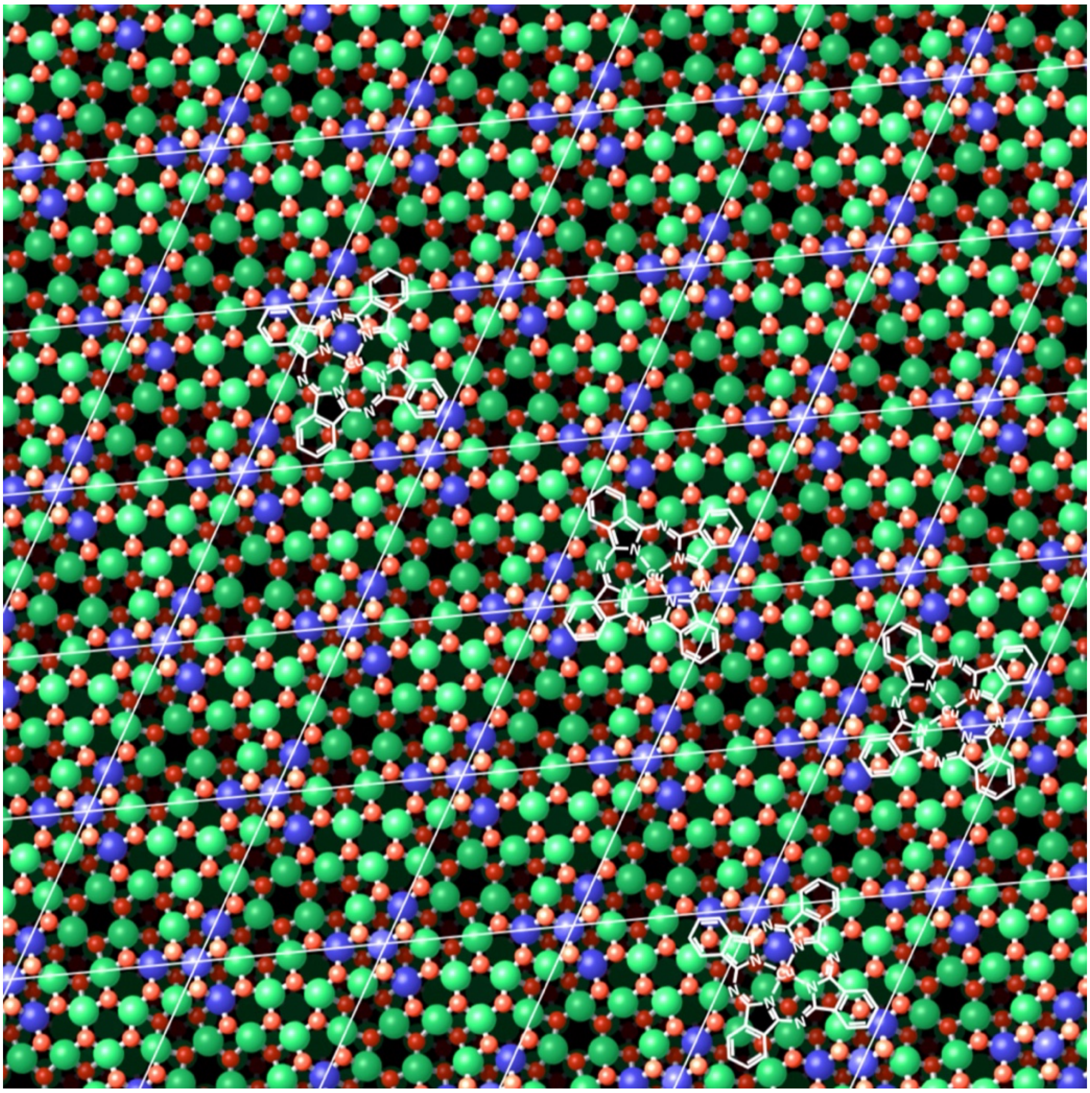

Figure S5: Identification of the adsorption site of the CuPc molecules. The position of the molecules with respect to the grid was determined from the experimental images in Fig. S4; here it is shown with respect to the structure model of the surface.

- **STM images and FFT**

Figure S6 displays 80 nm large STM images together with their Fast Fourier Transforms (FFT) of the bare $In_2O_3(111)$ as well as the (2 × 2) superstructure formed by 0.75 ML CuPc, and the (1 × 1) structure formed by 1 ML CuPc, respectively.

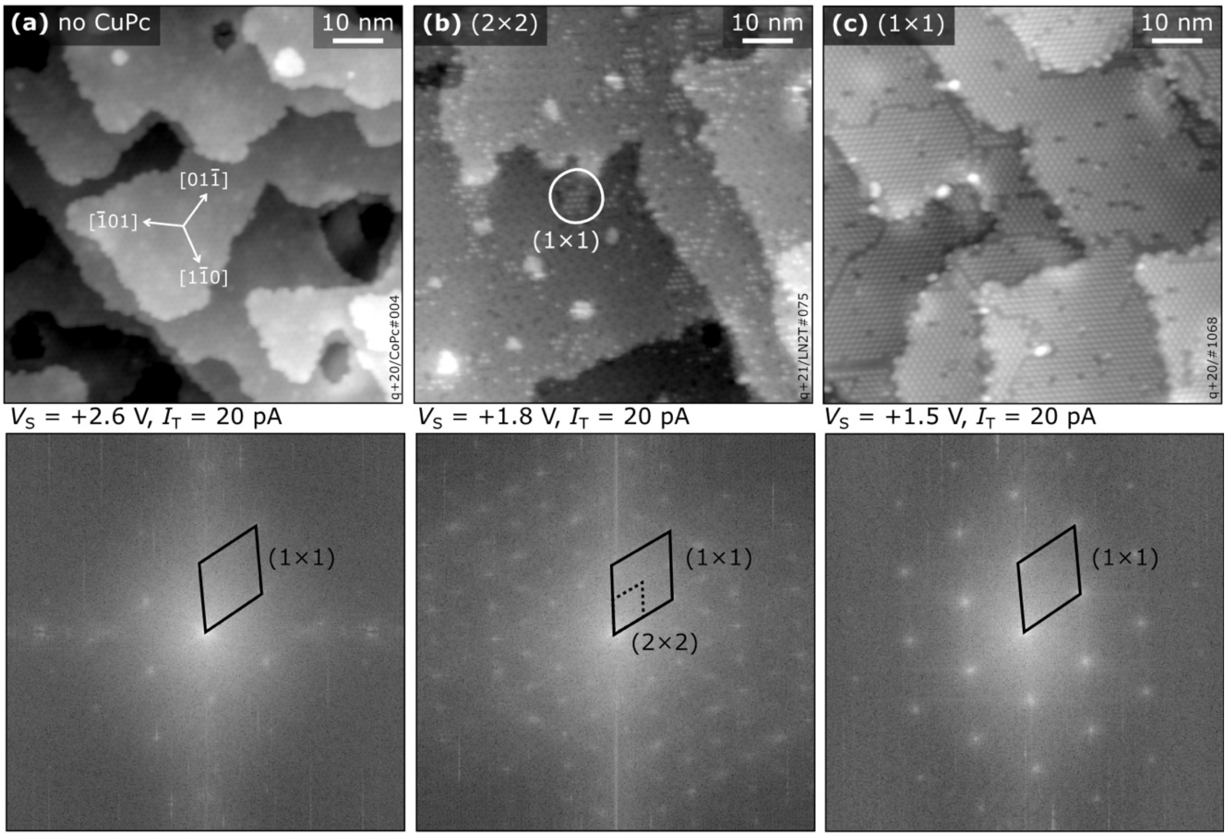

Figure S6: Overview STM images (80 nm) and their Fourier transforms (displayed with a logarithmic grayscale). (a) bare $In_2O_3(111)$ with some water impurities, (b) CuPc (2 × 2) at a coverage of 0.75 ML, and (c) CuPc (1× 1) at full monolayer coverage. (a,c) were acquired at 4.7 K, (b) at 80 K.

- **Isolated molecules and chains**

At low coverages, individual CuPc and also short chains are randomly distributed on the terraces of the substrate and without a preference for defects or step edges, see Figure S7.

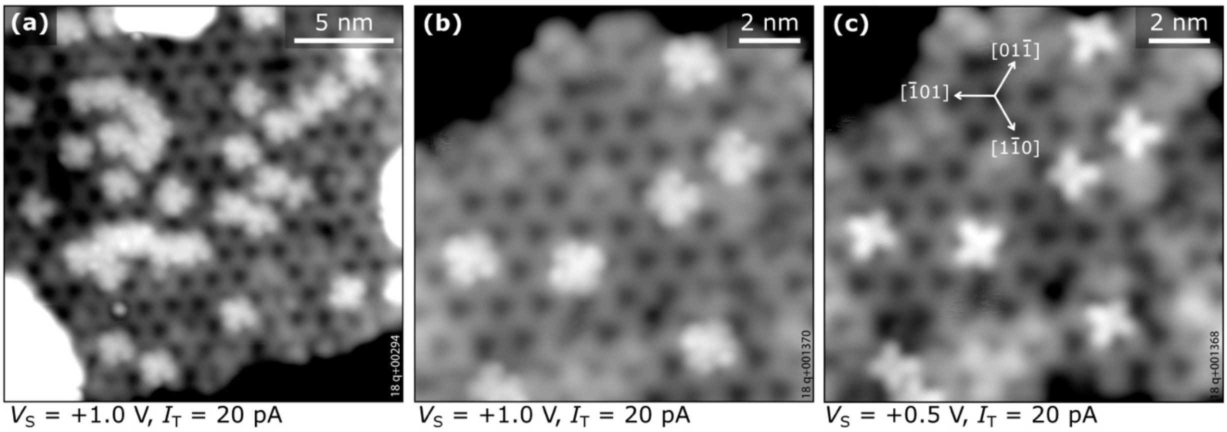

Figure S7: Overview of CuPc on $In_2O_3(111)$ for (a) a mixture of isolated molecules and short chains, and (b) isolated molecules.

The 1-dimensional chains found low coverages, see Figure S8, can align in two directions for each orientation of CuPc on $In_2O_3(111)$. The substrate atoms between the molecules is clearly different for these two orientations, see Figure S9. By evaluating 148 chains ranging from 2–7

molecules per chain (and excluding zigzag chains), a clear preference (≈82%) for chains with the In(5c)-terminated surface region between the molecules is observed (green lines in Fig. S9).

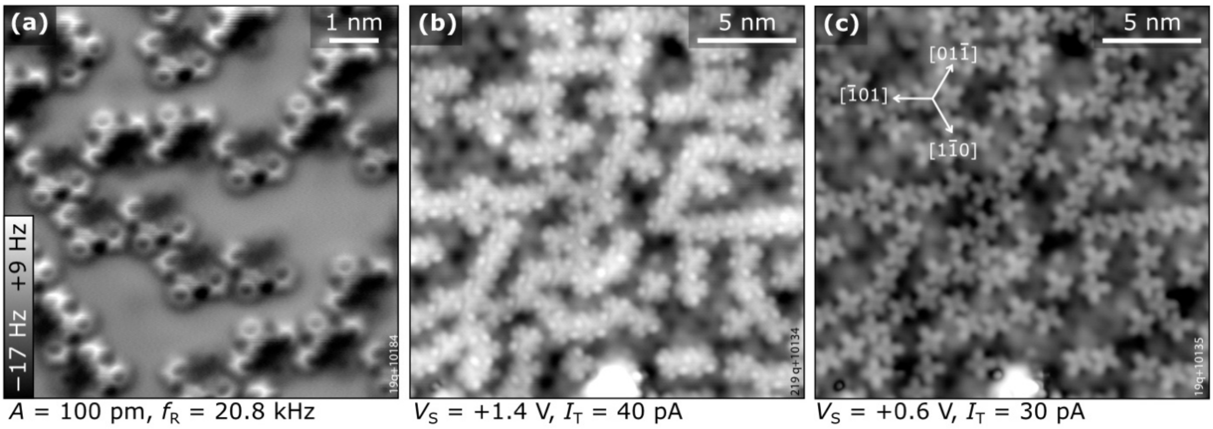

Figure S8: (2 × 2): CuPc chains on $In_2O_3(111)$.

- **Overview on all structures**

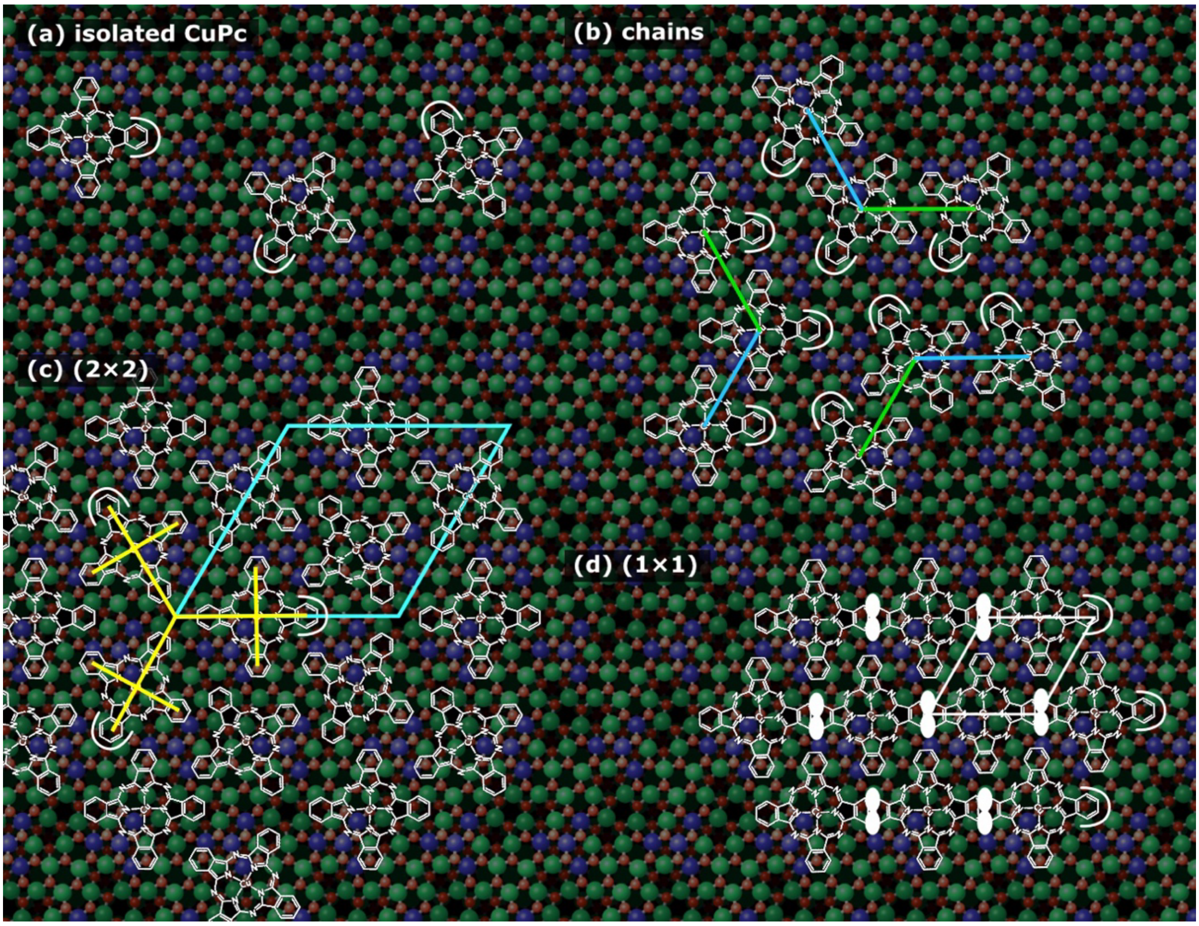

Figure S9: Schematic of all observed arrangements of CuPc on $In_2O_3(111)$ as function of coverage. (a) Isolated molecules in three orientations. (b) CuPc chains showing the differently coordinated surface atoms between the molecules of differently oriented chains (6-fold coordinated In in blue and 5-fold coordinated In in green). (c) (2 × 2) structure at 0.75 ML CuPc, with the (2 × 2) periodicity marked in cyan. (d) (1 × 1) structure at 1 ML of CuPc. The overlapping benzene rings are highlighted by white ovals. The white curves around the benzene rings label the isoindole groups tilted towards the surface.

- **(2 × 2) structure**

The (2 × 2) structure of CuPc on $In_2O_3(111)$ is visually reminiscent of the kagome lattice when imaged at bias voltages around +2.4 V, albeit with a lower symmetry (no mirror symmetry). Figure S10 displays a schematic of the kagome-like lattice and an STM image for comparison.

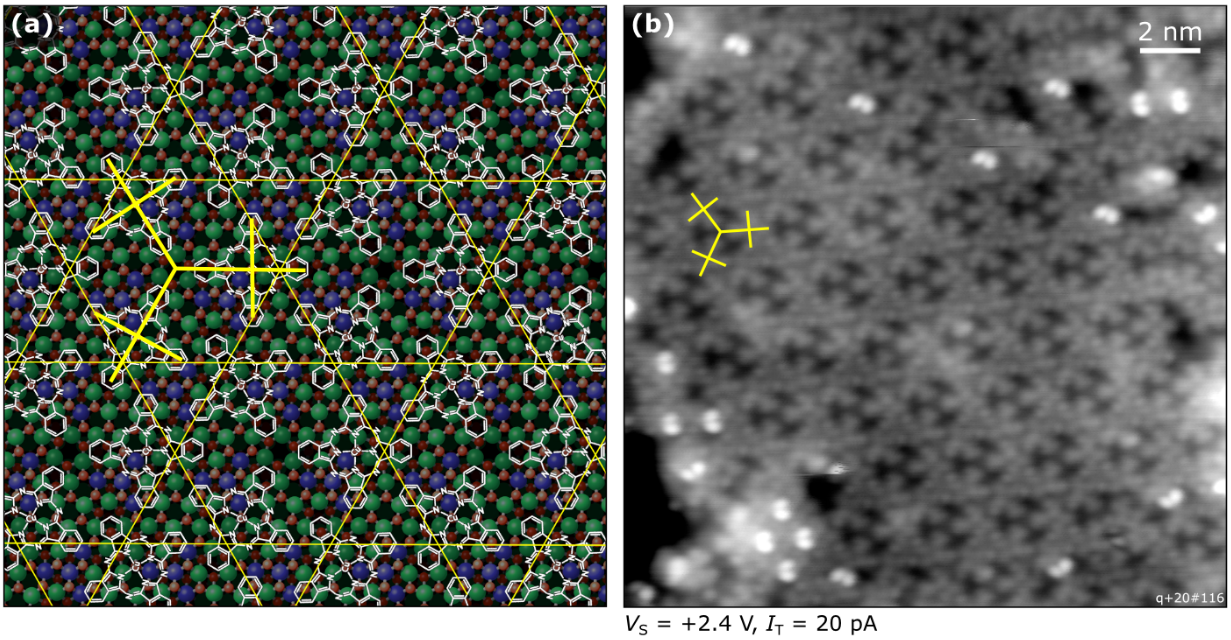

Figure S10: Structure model and kagome-like appearance of the (2 × 2) structure formed by 0.75 ML CuPc on $In_2O_3(111)$.

- **STM: Adsorption site within the (1 × 1) structure**

The adsorption site of the CuPc molecules in the densely packed (1 × 1) structure was determined from surface preparations with coverages between 0.75 and 1 ML, i.e., where both the (2 × 2) and (1 × 1) structure coexist. Examples of such surfaces are presented in Figure S11.

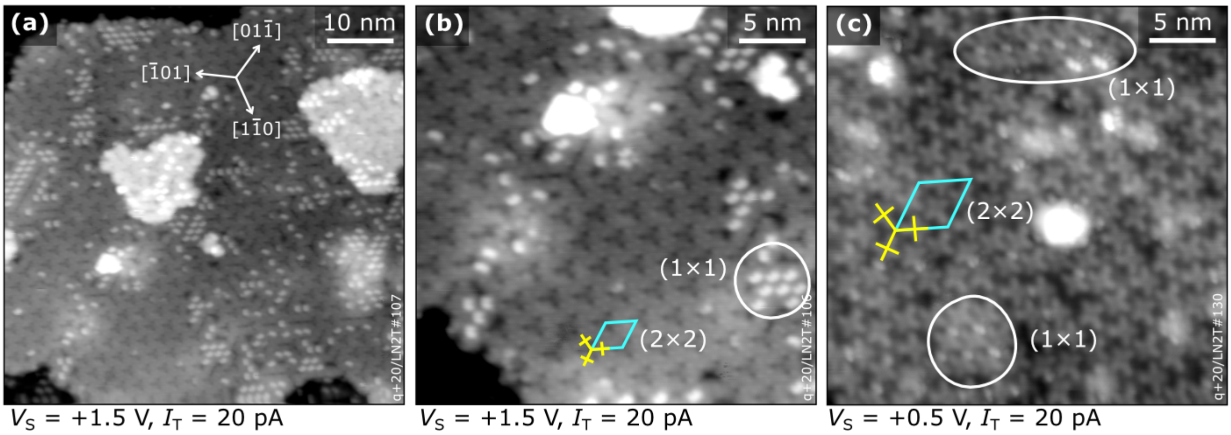

Figure S11: Mixture of the (2 × 2) and (1 × 1) structures. The (1 × 1) areas can be easily discerned due to the up-bent overlap regions, appearing as bright, elongated dots.

2. Computational

- **Comparison of flat versus tilted adsorption geometry (PBE)**

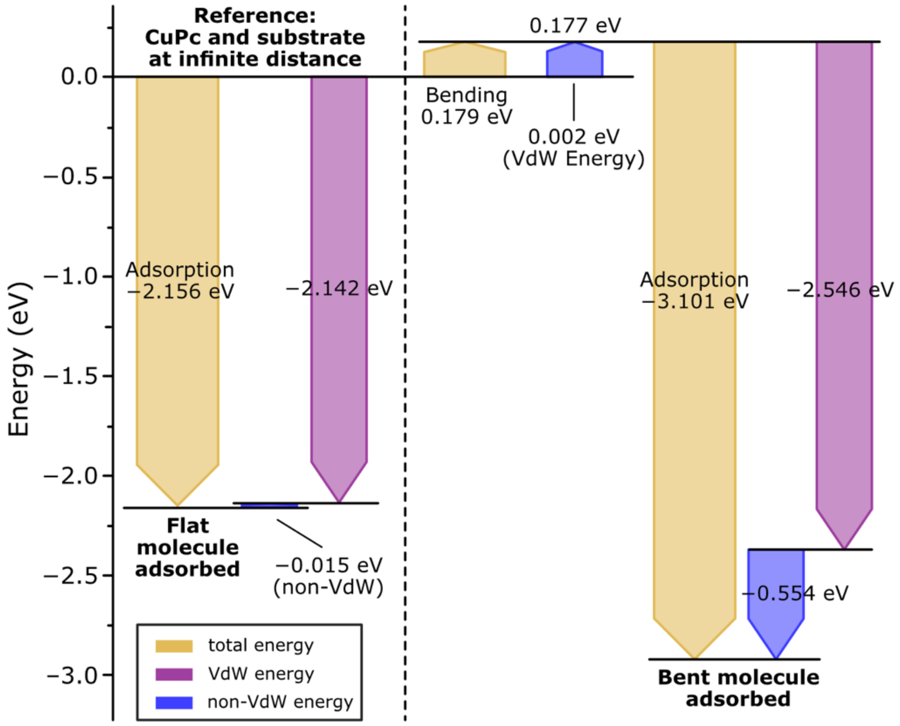

Figure S12: Comparison of the adsorption energy for a hypothetically flat CuPc with the experimentally observed tilted adsorption geometry. An increase in VdW energy is observed when the molecules is allowed to relax and tilts to follow the topography of the surface.

- **Parameter file used for the probe particle model**

```
probeType     54     # atom type of ProbeParticle (to choose L-J potential),
                     e.g., 8 for CO, 54 for Xe
charge -0.05  # effective charge of probe particle [e]
klat          1.7    # [N/m] harmonic spring potential (x,y) components, x,y is
                     bending stiffness
krad          20.00  # [N/m] harmonic spring potential R component, R is the
                     particle-tip bond-length stiffness
r0Probe       0.0    0.0    4.00   # [Å] equilibrium position of probe particle
                                   (x,y,R) components, R is bond length, x,y
                                   introduce tip asymmetry
PBC           True   # Periodic boundary conditions? [True/False]
gridA         21.45192417        12.38528771        0.00000000
gridB         -0.00001170        24.77057542        0.00000000
gridC         0.00000000         0.00000000         50.00000000
scanMin       0.0      0.0      15.0      # start of scanning (x,y,z)
scanMax       100.0    100.0    25.0      # end of scanning (x,y,z)
Amplitude     1.0    # [Å] oscillation amplitude for conversion Fz->df
scanTilt      0.0      0.0      0.0
```

```
tiltedScan    False
```

References

[1] H. Chen, M. A. Blatnik, C. L. Ritterhoff, I. Sokolović, F. Mirabella, G. Franceschi, M. Riva, M. Schmid, J. Čechal, B. Meyer, U. Diebold, M. Wagner, Water Structures Reveal Local Hydrophobicity on the In_2O_3(111) Surface, *ACS Nano,* 2022, **16**, 21163–21173. DOI: 10.1021/acsnano.2c09115

[2] M. Wagner, S. Seiler, B. Meyer, L. A. Boatner, M. Schmid, and U. Diebold. Reducing the In_2O_3(111) surface results in ordered indium adatoms. *Adv. Mater. Interfaces,* 2014, **1**, 1400289. DOI: 10.1002/admi.201400289

[3] M. Wagner, B. Meyer, M. Setvin, M. Schmid, and U. Diebold. Direct assessment of the acidity of individual surface hydroxyls, *Nature,* 2021, **592**, 722–725. DOI: 10.1038/s41586-021-03432-3
